# Single-Cell Electrical Stimulation Using CMOS-Based High-Density Microelectrode Arrays

**DOI:** 10.3389/fnins.2019.00208

**Published:** 2019-03-13

**Authors:** Silvia Ronchi, Michele Fiscella, Camilla Marchetti, Vijay Viswam, Jan Müller, Urs Frey, Andreas Hierlemann

**Affiliations:** ^1^Department of Biosystems Science and Engineering, ETH Zürich, Basel, Switzerland; ^2^MaxWell Biosystems AG, Basel, Switzerland

**Keywords:** HD-MEA, voltage stimulation, current stimulation, single-cell stimulation, axon initial segment

## Abstract

Non-invasive electrical stimulation can be used to study and control neural activity in the brain or to alleviate somatosensory dysfunctions. One intriguing prospect is to precisely stimulate individual targeted neurons. Here, we investigated single-neuron current and voltage stimulation *in vitro* using high-density microelectrode arrays featuring 26,400 bidirectional electrodes at a pitch of 17.5 μm and an electrode area of 5 × 9 μm^2^. We determined optimal waveforms, amplitudes and durations for both stimulation modes. Owing to the high spatial resolution of our arrays and the close proximity of the electrodes to the respective neurons, we were able to stimulate the axon initial segments (AIS) with charges of less than 2 pC. This resulted in minimal artifact production and reliable readout of stimulation efficiency directly at the soma of the stimulated cell. Stimulation signals as low as 70 mV or 100 nA, with pulse durations as short as 18 μs, yielded measurable action potential initiation and propagation. We found that the required stimulation signal amplitudes decreased with cell growth and development and that stimulation efficiency did not improve at higher electric fields generated by simultaneous multi-electrode stimulation.

## Introduction

Electrical stimulation (Rattay, 1999; Merrill, 2010) is a consolidated technique that has been widely used to study neuronal networks (Kumar et al., 2016; Wülfing et al., 2018), to treat brain diseases (Perlmutter and Mink, 2006; Benabid et al., 2009) and somatosensory dysfunctions (Brindley and Lewin, 1968; Shannon, 1983, 1985; Sekirnjak et al., 2008; Tsai et al., 2012; Grosberg et al., 2017; Greenberg et al., 2018; Fan et al., 2019), and to enhance moto-rehabilitation (Raspopovic et al., 2014; Armenta Salas et al., 2018). Electrical stimulation was combined with prosthetic implants in a variety of *in vivo* applications (Woodson et al., 2009; Dagnelie, 2012). For example, epiretinal implants feature electrodes that deliver an electric signal to neurons located in the retina, in close proximity to the optic nerve. The purpose of eye implants is to artificially substitute non-functional retina layers that fail to transduce light into electrical signals for the brain (Brindley and Lewin, 1968; Sekirnjak et al., 2008; Tsai et al., 2012; Grosberg et al., 2017; Fan et al., 2019). Electrical stimulation is employed as well in cochlear implants, where electrodes are used for hearing restoration by stimulating specific cochlear areas depending on sound frequency (Shannon, 1983, 1985; Greenberg et al., 2018). Neural stimulation has been used in the field of prosthesis embodiment for paralyzed patients to restore sensations in upper and lower limbs (Raspopovic et al., 2014; Armenta Salas et al., 2018). Furthermore, deep brain electrical stimulation of the subthalamic nucleus is used in Parkinson’s treatment (Perlmutter and Mink, 2006; Benabid et al., 2009) to reliably suppress and control the patients’ tremor.

Although a large variety of electrical stimulation-based prostheses exists, a major limitation of these devices is their low spatial resolution in delivering stimulation signals and the difficulty to locally constrain the electrical field to attain accurate and precise stimulation of preferably individual single cells. Indeed, blurred images, low sound resolution, inaccurate proprioceptive sensations, and adverse neurocognitive effects may be the results of imprecise electrical stimulation. The described shortcomings motivated us to explore stimulation parameters and regimes and to develop methods for accurate and precise charge injection. *In vitro* technologies enable to explore a large set of parameters to electrically stimulate neurons in cultures and 3D tissues or slices. Results and findings of *in vitro* studies of electrical stimulation can potentially be translated and optimized for *in vivo* applications (e.g., epiretinal implants and cochlear implants).

*In vitro* high-density microelectrode arrays (HD-MEAs) facilitate electrical-signal readout and stimulation of multiple neurons simultaneously at high-spatiotemporal resolution (Obien et al., 2015). Traditional microelectrode arrays have been used since 1970s (Thomas et al., 1972) for extracellular electrophysiology. Several studies have been carried out to investigate electrical stimulation parameters in neuronal cultures with the aim to find the most efficient way to elicit neuronal responses (Wagenaar et al., 2004; Ahmadian et al., 2011; Grosberg et al., 2017). Although the principles of electrical stimulation have been established, the large electrode size and pitch did not allow to perform stimulation at subcellular resolution and to demonstrate reliable single-neuron targeting.

The introduction of HD-MEAs in complementary-metal-oxide-semiconductor (CMOS) technology for *in vitro* applications (Eversmann et al., 2003; Berdondini et al., 2009; Frey et al., 2010; Ballini et al., 2014; Bertotti et al., 2014; Viswam et al., 2016; Tsai et al., 2017) enabled to obtain high spatial resolution and a large overall sensing area. Hundreds of researchers worldwide at universities, research institutes and pharmaceutical industries are currently using different CMOS-based HD-MEAs for their studies. CMOS-based HD-MEAs are also commercially available from several suppliers, including Multichannel Systems (Germany), 3Brain (Switzerland) or MaxWell Biosystems (Switzerland). With the advent of neurons derived from human induced pluripotent stem cells (hiPSCs), the interest in HD-MEAs is rapidly growing, as such devices are suitable to assess hiPSC functionality of healthy and disease phenotypes. In this study, we used a 26,400-electrode CMOS chip (Ballini et al., 2014), with a sensing area of 3.85 × 2.10 mm^2^, an electrode pitch of 17.5 μm and an electrode size of 5 × 9 μm^2^, which provided subcellular resolution for readout and stimulation. The device enabled targeting of the axon initial segment (AIS) for stimulation, which was demonstrated to ensure efficient and accurate stimulation (Radivojevic et al., 2016; Bakkum et al., 2018), and the device enabled signal readout upon stimulation in direct proximity to the cell soma of the very same cell.

An important issue with electrical stimulation through microelectrodes in densely packed arrays is the so-called “stimulation artifact,” which is characterized by saturation of the recording amplifiers that are connected to the stimulation electrode itself and the surrounding electrodes during hundreds of microseconds or longer. This saturation is a consequence of the large stimulation signal amplitudes, ∼50–100 mV, while the readout amplifiers feature microVolt sensitivity. Different from other approaches (Hottowy et al., 2012), we did not aim at recovering the signal on the stimulation electrode itself, as the large density of electrodes, present in our array, allows for recording from different electrodes, still spanning under the same neuron of interest. Moreover, we did not use any strategy to suppress artifacts in this study, as our interest was to compare the different stimulation strategies and parameters also with respect to artifact generation. Electrodes at a distance of only 17.5 μm from the stimulation electrode were already available for readout in all cases, partially due to the comparably low stimulation-signal amplitudes that we could afford owing to accurate targeting of the stimulation-sensitive AIS (Radivojevic et al., 2016). The artifact depends on the stimulation signal amplitude, the applied waveform, and its duration. Therefore it is crucial to identify the stimulation signal that produces the lowest artifact while still reliably inducing an action potential (AP).

This study was targeted at finding optimal stimulation modalities, i.e., to achieve the most efficient stimulation of neurons with our HD-MEAs at minimal artifacts, by comparing different stimulation waveforms, amplitudes and durations, both in voltage and current mode. We used biphasic and monophasic rectangular waveforms for stimulation in voltage mode (Wagenaar et al., 2004), and charge-balanced biphasic and triphasic rectangular waveforms for stimulation in current mode (Wagenaar et al., 2004; Hottowy et al., 2012; Grosberg et al., 2017). We compared the efficacy of the voltage and current stimulation regimes, characterized the influence of the electrode impedance, and measured stimulability during cell growth and development in culture. Finally, we simulated and tested different multi-electrode configurations and compared the obtained results.

## Materials and Methods

### Animal Use

All experimental protocols were approved by the Basel Stadt veterinary office according to Swiss federal laws on animal welfare and were carried out in accordance with the approved guidelines.

### High-Density Microelectrode Arrays

A CMOS-based HD-MEA (Ballini et al., 2014) was used for *in vitro* stimulation and recording. The device features 26,400 Pt electrodes (each 5 × 9 μm^2^ at a pitch of 17.5 μm) occupying a sensing area of 3.85 × 2.10 mm^2^. The HD-MEA system includes 1,024 configurable readout channels that can be used to record simultaneously. The readout-channel noise is 2.4 μV_rms_ in the band between 300 Hz and 10 kHz and 5.4 μV_rms_ in the band between 1 and 300 Hz. The readout channels’ gain is programmable up to 78 dB, depending on the application. Additionally, the device features 32 stimulation units that can be used in both, current or voltage mode. The sampling frequency is 20 kSamples/s, and the overall power consumption is 75 mW. Gold bond wires connect the chips to printed circuit boards (PCBs) and are protected from saline solutions (e.g., culture medium) using epoxy (Epo-Tek 353ND, 35ND-T, Epoxy Technology Inc., Billerica, MA, United States). The electrodes were coated with Pt-black, the chips were then sterilized for 40 min in 70% ethanol and rinsed 3 times with deionized (DI) water before every cell plating.

### Platinum Black Deposition

A porous Pt-black layer was deposited on the electrodes to increase the surface area and decrease the electrode impedance, which improves the signal-to-noise ratio (SNR) of recorded signals. A 2 mL solution of chloroplatinic acid hexahydrate (7 mM, Sigma-Aldrich, Saint Louis, MO, United States) and lead acetate (0.3 mM, Honeywell, Morris Plains, NJ, United States) in DI water was pipetted onto the exposed region of the HD-MEA chip. A Pt reference electrode was immersed in the solution and current of 550 μA was applied to the array electrodes for 1.30 min.

### Cell Cultures

Prior to culturing cells, the HD-MEA electrode area was treated with 20 μL of 0.05% (v/v) poly(ethyleneimine) (Sigma-Aldrich) in borate buffer (Thermo Fisher Scientific, Waltham, MA, United States) at 8.5 pH, for 40 min at room temperature. This step improves cell adhesion and makes the substrate more hydrophilic. We rinsed the chips three times with DI water. Next, we added 8 μL of 0.02 mg ml^−1^ laminin (Sigma-Aldrich) in Neurobasal medium (Gibco, Thermo Fisher Scientific) to support the growth and differentiation of the cells. The chips were incubated with laminin for 30 min at 37°C. During this time, we dissociated cortices of Wistar rats at embryonic day 18 in trypsin with 0.25% EDTA (Gibco). After 20 min of digestion, the cortices were washed twice with plating medium, then triturated, and the cells were counted. We counted with a hemocytometer by diluting the cells in 0.4% Trypan blue stain solution (Gibco). We then seeded between 15,000 and 25,000 cells over an active area of approx. 8 mm^2^. The chips were afterward incubated at 37°C for 30 min before adding 1.5 mL of plating medium. The plating medium consisted of 450 mL Neurobasal (Invitrogen, Carlsbad, CA, United States), 50 mL horse serum (HyClone, Thermo Fisher Scientific), 1.25 mL Glutamax (Invitrogen), and 10 mL B-27 (Invitrogen). After 76 h, we changed 50% of the plating medium to growth medium, which consisted of 450 mL D-MEM (Invitrogen), 50 mL Horse Serum (HyClone), 1.25 mL Glutamax (Invitrogen) and 5 mL sodium pyruvate (Invitrogen). The procedure was repeated twice a week. The chips were kept inside an incubator at 37°C and 5% CO_2_. Every chip was equipped with a lid, and additional DI water in a 35-mm-Ø petri-dish was added to prevent evaporation. All the experiments were conducted between days *in vitro* (DIVs) 10 and 30.

### Microscopy and Stainings

We used NeuroFluor NeuO (Stemcell Technologies, Vancouver, Canada) live staining to locate neurons on the array before the stimulation experiments. The cells were incubated for 1 h at 37°C with 2 mL growth medium containing 0.15% NeuO. The chips were then washed 2 times with growth medium.

We also performed neuron fixation after stimulation experiments by using 4% paraformaldehyde (Life Technologies, Thermo Fisher Scientific). Sample permeabilization and blocking of non-specific antibody binding were done using a PBS 1× solution containing: 10% normal donkey serum (NDS) (Sigma-Aldrich), 1% bovine serum albumin (BSA) (Sigma-Aldrich), 0.02% Na-Az (Sigma-Aldrich), 0.5% Triton X (Sigma-Aldrich). Primary and secondary antibodies were diluted in a PBS 1× solution containing: 3% normal donkey serum (NDS), 1% bovine serum albumin (BSA), 0.02% Na-Az, 0.5% Triton X. We used antibodies against MAP2 (Abcam, Cambridge, United Kingdom), Ankyrin G (Santa Cruz Biotechnology, Dallas, TX, United States), and the fluorescent dye Hoechst (Invitrogen) to stain neurons, axonal initial segments (AIS), and nuclei. We imaged cells on the HD-MEA chip with a Nikon NiE upright confocal microscope, with a Yokogawa W1 spinning disk scan head, 6 laser lines and a fluorescence recovery after photobleaching (FRAP) unit.

### Stimulation and Data Analysis

Electrical stimulation was controlled via an on-chip digital analog converter (DAC) and software programmable through a Python application programming interface (API).

We used both, voltage and current stimulation modalities. In both, a charge is applied to the stimulation electrode. Ideally, only charge redistribution in the double-layer capacitor, formed at the electrode/electrolyte interface, occurs and charge transfer and redox reactions involving electron transfer at the electrode surface (Faradaic processes) are avoided. Using current stimulation, the charge can directly be controlled, while the voltage may assume large values depending on the specific current path. High electrode voltages may produce unwanted electrochemistry (Faradaic processes), tissue damage, or electrode degradation. In the case of voltage stimulation, one can control the voltage, while the injected current depends on the electrode impedance (Supplementary Figure 1), which may vary considerably due to fabrication variation or aging. Precisely controlling the applied voltage helps to prevent electrolysis, which may occur outside the water window and may damage the electrodes or cause cell death.

We used a randomized voltage stimulation protocol including four different waveforms: biphasic cathodic-anodic, biphasic anodic-cathodic, monophasic anodic, monophasic cathodic, see also Figure 2A (Wagenaar et al., 2004). The protocol included four durations of 50, 100, 150, and 200 μs per phase and six stimulation signal amplitudes (40, 80, 120, 160, 200, and 240 mV peak-to-peak). For current stimulation, we applied a randomized protocol of two waveforms, biphasic anodic-cathodic and triphasic anodic-cathodic-anodic, both charge balanced (Grosberg et al., 2017), five durations of 10, 15, 18, 20, and 50 μs per phase, and eight stimulation signal amplitudes (42, 63, 84, 105, 126, 147, 168, and 189 nA). Every individually shaped stimulation pulse of both modalities was repeated 30 times during the entire protocol in a randomized way in order not to evoke neuronal plasticity processes. The stimulation frequency was 1 Hz for both modalities, as stimulation in the frequency band between 0.2 and 1 Hz was reported not to entail significant changes in the AIS position (Grubb and Burrone, 2011). We selected 1 Hz, the upper bound, to limit overall time needed for the stimulation experiments.

A custom-made software was used to visualize and record the extracellular signals from the electrodes. The extracellular action potential (EAP) spatial distribution or “electrical footprint” of a neuron, which is the voltage-signal distribution over the multiple electrodes, was reconstructed using spike sorting algorithms (UltraMegaSort, Hill et al., 2011). The software identifies the spikes with a threshold of 4.5 times the standard deviation of the noise. Using this software, we then could identify and select the stimulation electrodes based on the EAP amplitudes.

The collected data was analyzed in MATLAB. To verify the presence of an evoked APs, we set a threshold of four times the standard deviation of the noise, together with a temporal window of 1.5 ms. An automatic script registered the EAPs for the 30 repetitions of every sent waveform, and rendered a visual record that could be inspected to verify the counting. Cases where the artifact partially covered the EAPs were classified as “missing EAP” during automatic registration. In these cases, we applied visual inspection and manual correction as appropriate.

### Impedance Characterization

To characterize electrode impedances, we applied a readout gain of 2 and 20 repetitions of a biphasic anodic-cathodic current stimulation pulse with a duration of 1 ms per phase and an amplitude of 140 nA to bright Pt electrodes and with a duration of 2.5 ms per phase and an amplitude of 560 nA to Pt-black electrodes. Such low gain in the readout channels avoided DC voltage saturation of the stimulation channel. The lower waveform durations and amplitudes avoided channel saturation in case of bright Pt, because of the higher impedance.

To determine the electrode impedance, we fitted the voltage readout on the stimulation electrode with equations derived from the Gouy–Chapman–Stern model of an electrode (Franks et al., 2005) (Supplementary Figure 2). We added a constant equivalent input impedance *Z*_in_ for the recording channel input impedance in parallel to the electrode equivalent circuit. The electrode equivalent circuit had two unknown values, the charge transfer resistance, *R*_ct_, and the double layer capacitance, *C*_dl_. These values were computed by fitting the obtained experimental data in MATLAB using the following equation:

$$V(t)=\frac{I_{\text{stim}}R_{\text{ct}}\left(1-e^{-\frac{t}{C_{\text{dl}}R_{\text{ct}}}}\right)}{1+\frac{R_{\text{ct}}}{Z_{\text{in}}}\left(1-e^{-\frac{t}{C_{\text{dl}}R_{\text{ct}}}}\right)}$$

In this equation *V* is the readout voltage and *I*_stim_ the applied stimulation current. We defined initial values for *C*_dl_ and *R*_ct_ by referring to values reported in literature (Héduit et al., 1996; Franks et al., 2005; Oldham, 2008; Joye et al., 2009; Sharma and Bhatti, 2010).

To confirm the simulation results with a larger number of electrodes, we applied a sine-wave voltage stimulation on the reference electrode surrounding the array and recorded the corresponding signals through electrode sets of the array. The sine wave had a frequency of 1 kHz and an amplitude of 50 mV_pp_ (peak-to-peak). The recording channels and circuits are characterized by a finite and constant input impedance, which should ideally be high for neural applications and higher than the electrode impedance to ensure signal integrity (Obien et al., 2015). We compared the obtained electrode impedances with the input impedances of the recording channels for the whole array to characterize impedance homogeneity across the array. We also performed impedance measurements on three stimulation electrodes in PBS, before and after conducting a full set of stimulation experiments in current and voltage mode. After the experiment, the impedance varied in average by 0.07 nF (∼5% of the average electrode impedance value for Pt black).

### COMSOL Simulations

We simulated the stimulation pulse extension across the array of the used multi-electrode configurations in *COMSOL Multiphysics 5.3a*. The model includes 4 main components (libraries): geometry, materials, electric currents, and mesh. For the geometry, we used a configuration of 36 planar electrodes, with a 5 × 9 μm^2^ surface area for each electrode and a pitch of 17.5 μm, which is consistent with the HD-MEA electrode characteristics. An external block of 500 × 500 × 100 μmł was added as the electrolyte solution. Four reference electrodes (dimensions of 5 × 245 μm^2^) were placed around the electrode array. The electrodes were simulated assuming platinum as electrode material, while the electrolyte solution was a saline solution with an electrical conductivity of 0.7 S m^−1^. For the electrical characterization, we simulated voltage stimulations. We used a biphasic anodic-cathodic waveform with an amplitude of 100 mV and a duration of 100 μs per phase. The electric-current library was used to simulate the voltage and electric field distributions upon voltage stimulation.

### Multi-Electrode Stimulation

To implement multi-electrode stimulations in voltage mode, we used a custom-made Python script to control two DACs to stimulate two electrodes at the same time. In a first configuration, we applied a biphasic waveform on one electrode and the same waveform but with opposite sign on a neighboring electrode. This configuration limited the charge flow across the array, which decreased the artifact extension and spreading. In a second configuration, we applied a biphasic waveform to one electrode and connected the neighboring electrode to ground with the intention to limit the electric field and artifact extension. External reference electrodes remained always connected.

### Availability of Materials

Adapted MATLAB scripts and COMSOL model scripts, used for the analysis of the data in Figure 2–6 are available at the following repository: ElectricalStimulation (link: https://github.com/sronchi/ElectricalStimulation). Moreover, we can provide raw data sets (total of 10 TB) at reasonable request.

## Results

### Artifacts of Current and Voltage Stimulation

**FIGURE 1:**
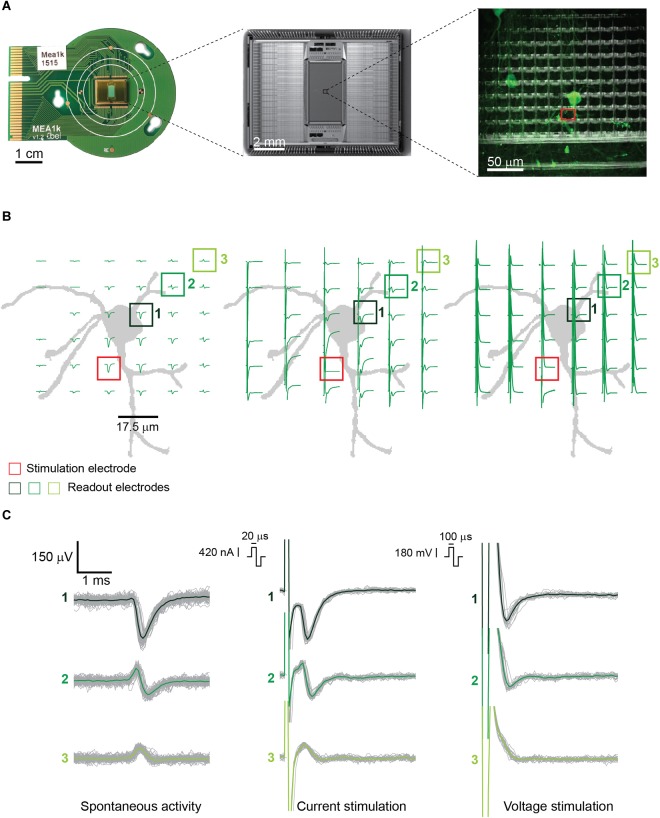
**(A)** (from left to right) PCB-mounted HD-MEA chip, photomicrograph of the chip, and enlargement of a subsection of the array including about 100 electrodes with live-stained neurons highlighted in green. The stimulation electrode used in panel **B** is highlighted in red. The picture was taken using an immersion 60**×**-magnification lens at the periphery of the array, where the cell density is lower. **(B)** Illustration of the neuron, labeled in panel **A**, and corresponding electrode locations, with superimposed measured signals. (Left) Spontaneous EAPs obtained after spike sorting. (Center) EAPs after current stimulation of the selected neuron. (Right) EAPs after voltage stimulation. Current stimulation entailed a biphasic anodic-cathodic waveform of 20 μs per phase. Voltage stimulation entailed a biphasic anodic-cathodic waveform of 100 μs per phase. The smallest stimulation signal amplitudes that still evoked APs 27 times during 30 repetitions (90% efficiency) were used. **(C)** Signals from three of the readout electrodes in panels **A** and **B** (numbered boxes). (Left) Extracellular signals recorded during spontaneous neuronal activity composed of >100 detected EAPs. (Center and right) Extracellular signals recorded from the same electrodes during 30 repetitions of current and voltage stimulation.

A major limitation of any electrical stimulation is the resulting artifact, which obscures the EAP readout likewise in current and voltage modes. To compare artifacts generated during voltage and current stimulation of cortical neurons, we plated ∼ 15,000 cortical neurons on the array and labeled them neurons with NeuroFluor NeuO live-staining (Figure 1A) to identify individual neurons. We determined the most suitable electrode and the smallest stimulation signal amplitude that could evoke EAPs with 90% success rate over 30 repetitions. We used biphasic waveforms in both modalities and compared cases with similar artifact shapes. The duration was set to 100 and 20 μs per phase for voltage and current stimulation, respectively. The different durations were a consequence of the high efficiency of the stimulation buffers in current mode, which showed a reliable charge injection for durations longer than 18 μs (Supplementary Figures 3, 4). In current mode, the stimulation buffers could deliver a sharp charge injection regardless of the electrode impedance. In voltage mode, however, the shortest efficient pulse duration was found to be >50 μs (Paragraph 3.2, Supplementary Figure 4), due to the different stimulation buffers’ design (Ballini et al., 2014).

Since the artifact duration is governed primarily by the stimulation pulse duration, shorter current pulses produced shorter, more easily detectable and distinguishable AP artifacts than the voltage pulses. This is shown in Figure 1B,C. For the latter, not only were the nearby extracellular APs obscured, but the artifact amplitudes were also significantly larger. In voltage mode, the evoked EAPs were shifted with respect to the baseline, in comparison to spontaneous EAP activity, so that they could not always be easily detected by using a negative amplitude threshold (four times the standard deviation of the noise) for spike detection. Only the neurons producing high-amplitude EAPs could be detected by using a negative voltage threshold while the other neurons were not used for analysis.

Owing to the high-density electrodes, the short current stimulation pulse enabled a signal readout and determination of stimulation success already on the next neighboring electrode, 17.5 μm away from the stimulation site (Figure 1B,C, center and right). Upon stimulating axonal compartments, like the AIS, it was possible to measure signals at the corresponding cell soma of the same neuron, which enabled to unambiguously determine stimulation success.

### Effect of Durations, Amplitudes and Waveforms, in Voltage and Current Stimulation Modes

**FIGURE 2:**
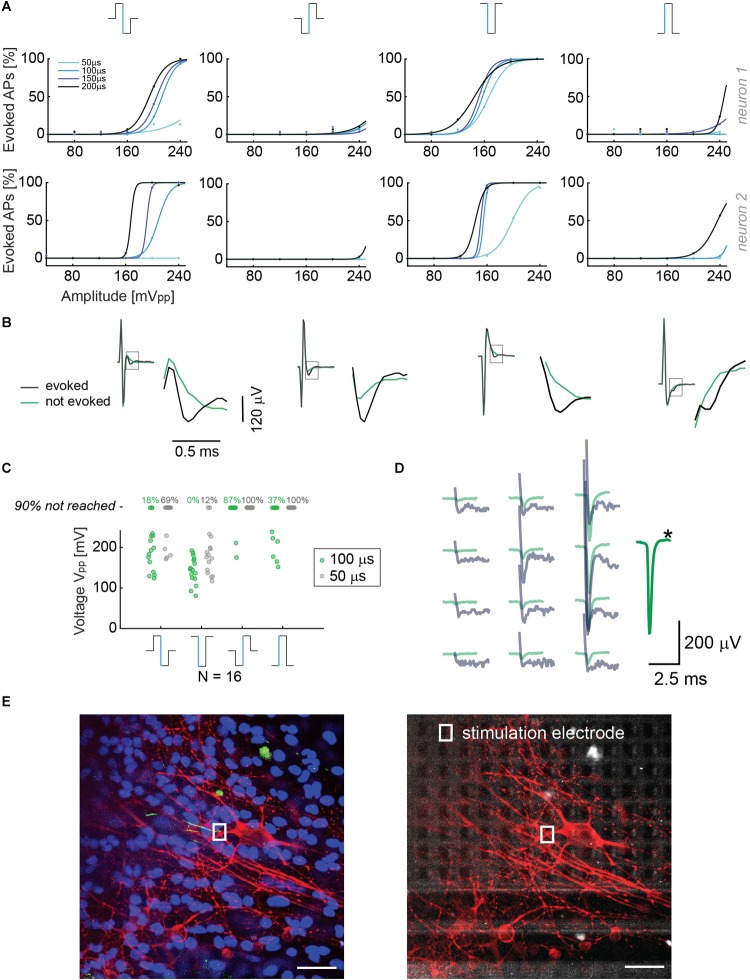
**(A)** Results from voltage stimulation of two neurons. At the top, the four different waveforms that were used are displayed. The figure shows that lower voltage amplitudes are sufficient to evoke APs when using monophasic cathodic and biphasic anodic-cathodic waveforms. A phase duration of 50 μs was less efficient than phase durations of 100, 150, and 200 μs. We applied 30 repetitions for every waveform, duration and amplitude in a randomized manner. **(B)** Recorded voltage signals including stimulation artifact for successful and non-successful voltage stimulations are displayed for the four stimulation waveforms in panel **A**. No measures were taken to suppress the artifact. The close-ups show the region, in which the neuron’s response eventually occurred. The voltage signal (artifact only) recorded during/after a stimulation that did not evoke an AP is displayed in green, voltage signals (artifact plus superimposed neuronal response) recorded during a successful stimulation are displayed in black. **(C)** Voltage stimulation results of 16 neurons. The points represent the smallest voltages (Vpp) that evoked APs in 90% of the stimulations during 30 repetitions; the signal duration was 100 μs per phase (green dots) or 50 μs per phase (gray dots). At the top, the percentages of failure in evoking APs upon using the 4 waveforms with amplitudes of up to 240 mV are given for the 2 different durations. **(D)** Overlay of the spike-sorted spontaneous activity of a neuron (green) and its response (gray) upon voltage stimulation through the electrode marked with a black star (^∗^). **(E)** Neuron stainings; nuclei were stained in blue (Hoechst), neuronal structures in red (anti-MAP2) and the AIS in green (Ankyrin G). The stimulation electrode is indicated with a rectangle (white). In the right picture, the electrode array is visible below the stimulated neurons. Scale bar: 35 μm.

Although we noticed that the HD-MEA produced smaller artifacts in current mode, we wanted to study the relevant parameters to efficiently evoke APs in both, current and voltage modes. Current stimulation is preferred and used for many *in vivo* applications (Raspopovic et al., 2014), due to the fact that the injected charge can be determined independently of the impedance. However, voltage stimulation offers the advantage to precisely control the voltage and avoid electrode or cell/tissue damage as a consequence of electrolysis (for a more detailed discussion see Section 2.6). In line with previous electrical-stimulation studies *in vitro* (Wagenaar et al., 2004; Hottowy et al., 2012; Grosberg et al., 2017), we investigated different parameters for both, current and voltage modes. Our strategy was to efficiently stimulate neurons in the region of the AIS and to then read out the corresponding evoked action potentials at the cell soma and several other locations, which was possible due to the availability of a large number of electrodes at high density, the small signal amplitudes needed to stimulate at the AIS, and the possibility to deliver short and efficient stimulation pulses (≥18 μs). For voltage stimulation, we used biphasic cathodic-anodic, biphasic anodic-cathodic, monophasic anodic and monophasic cathodic waveforms. The stimulation protocol included four durations for every waveform and six amplitudes in a randomized sequence (40, 80, 120, 160, 200, and 240 mV peak to peak). Neurons were stimulated between DIV 10 and 30. Stimulation electrodes were selected after identifying the spatial distribution or electrical footprint of extracellular EAPs of individual neurons. Electrodes recording the highest amplitudes of single-neuron action potentials were selected as stimulation electrodes, as they were presumably located under the neuronal compartments that are most sensitive to stimulation (Radivojevic et al., 2016; Bakkum et al., 2018). The compartment producing the largest-amplitude EAPs and being most sensitive to stimulation has been recently identified as the AIS (Radivojevic et al., 2016; Bakkum et al., 2018). The results are displayed in Figure 2A: The stimulation results of two different neurons upon applying four stimulation voltage waveforms with different amplitudes and waveform durations are shown. The monophasic cathodic waveform was found to be the most efficient waveform in evoking APs in the voltage mode, followed by the biphasic anodic-cathodic waveform. The monophasic anodic and the biphasic cathodic-anodic waveform featured lower efficiency. Another result we observed is that a phase duration of 50 μs was not sufficient to reliably evoke APs, whereas there was no major difference for phase durations longer than 100 μs. In Figure 2B, recorded voltage waveforms and close-ups of successful (AP was elicited, black) and unsuccessful (no AP, green) stimulations are shown to demonstrate how EAPs look like in the presence of stimulation artifacts. To consolidate the stimulation results in 2A, we repeated the randomized protocols with 16 additional neurons (Figure 2C). We determined the peak-to-peak voltage (V_pp_), which was needed to evoke APs in 90% of the stimulations over 30 repetitions for those cells. Two phase durations, 50 μs (gray) and 100 μs (green), were used and compared. At the top of the graph, the percentages of failure in evoking APs, while using the 4 waveforms up to a maximum amplitude of 240 mV are given for the 2 different durations. The obtained results confirmed the aforementioned low efficacy of 50 μs phase duration. For using a phase duration of 50 μs, one should, for a successful stimulation, deliver the same charge as for using 100 μs, but the settling time of the stimulation buffers and the electrode impedance imposed limits on the stimulation efficacy with such short phase durations (see Supplementary Figure 4).

To confirm that the selected neurons were effectively stimulated, we superimposed the “electrical footprints” of spontaneous activity after spike sorting (using UltraMegaSort) with the spatial distribution of stimulation-evoked EAPs. The spontaneous activity was recorded using a high-density block of electrodes in the region of interest during at least 1 min (>100 EAP). The superposition shows a spatial and temporal match of spontaneous and stimulation-triggered EAPs. However, the amplitudes of the superimposed EAPs are not so easy to compare as a consequence of the stimulation artifact (Figure 2D). A clear identification of the neuron could be performed by using its electrical “footprint,” the spatial distribution of extracellular APs in conjunction with upright confocal microscopy. Using these features and methods, we could prove that, indeed, the neuron of interest was stimulated in its perisomatic region as EAP readout of the very same neuron was possible, e.g., in an axonal branch nearby. Stimulation was very selective, and individual neurons could be stimulated without eliciting EAPs in neighboring neurons.

After execution of the electrical stimulation protocols, selected neurons were stained for correlating neuron morphologies with their EAP spatial distribution, recorded through the electrodes. In particular, we investigated which neuronal compartment was closest to the stimulation electrode. We observed that the most efficient stimulation electrode was located in close proximity to the AIS (green AnkyrinG staining), confirming previous reports (Radivojevic et al., 2016; Bakkum et al., 2018) (Figure 2E).

**FIGURE 3:**
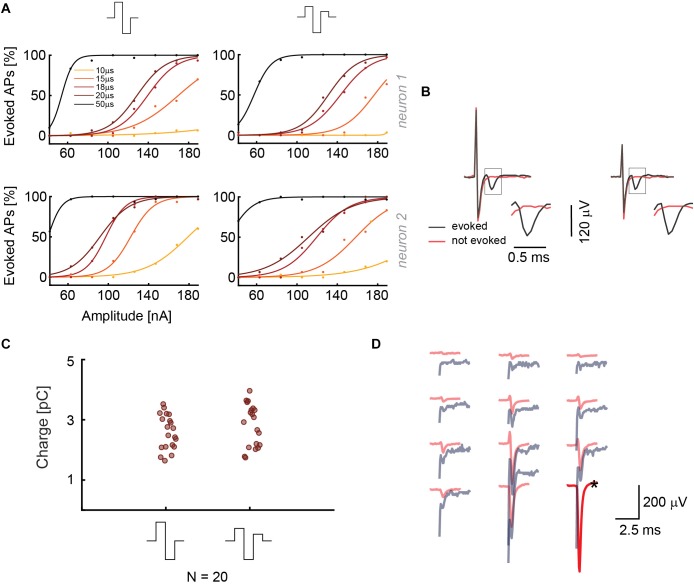
**(A)** Results from current stimulation of two neurons. At the top, the two waveforms that were used are displayed. The efficacy in evoking APs is almost the same for both waveforms. 30 repetitions were used for every waveform, phase duration and stimulation signal amplitude in a randomized manner. **(B)** Recorded voltage signals including stimulation artifacts for successful and non-successful current stimulations are displayed for the two stimulation waveforms in panel **A**. No measures were taken to suppress the artifact. The close-ups show the region, in which the neuron’s response eventually occurred. The voltage signal (artifact only) recorded during/after a stimulation that did not evoke an AP is displayed in red, voltage signals (artifact plus superimposed neuronal response), recorded during a successful stimulation, are displayed in black. **(C)** The current stimulation results of 20 neurons are displayed. The points represent the smallest charges that evoked APs in 90% of the stimulations during 30 repetitions; the signal duration was 20 μs per phase. **(D)** Overlay of the spike-sorted spontaneous activity of a neuron (red) and its response (gray) upon current stimulation through the electrode marked with a black star (^∗^).

In a second set of experiments, we used similar protocols for the current stimulation mode, where we investigated the effect of two different waveforms, namely biphasic anodic-cathodic, and triphasic anodic-cathodic-anodic (amplitude ratio 2:3:1). We used five phase durations and eight signal amplitudes in a randomized sequence (42, 63, 84, 105, 126, 147, 168, and 189 nA). Monophasic waveforms were not used to not compromise charge balancing. The results of two different stimulated neurons in Figure 3A show that the two waveforms provide similar efficiency in stimulating the targeted neurons for different phase durations and amplitudes. In Figure 3B, recorded voltage waveforms and close-ups of successful (AP was elicited, black) and unsuccessful (no AP, red) stimulations are shown to demonstrate how EAPs look like in the presence of stimulation artifacts. The results can be compared with panel 2B, showing the signals for the voltage stimulation mode. For the voltage stimulation mode, the AP always is superimposed on the artifact, whereas, in current mode, the evoked EAP is temporally more clearly separated from the artifact and much easier to detect. This better detectability is, to some extent, a consequence of the shorter duration of the stimulation signal in the current mode. To consolidate the stimulation results, we repeated the same randomized protocol with 20 additional neurons with a phase duration of 20 μs. Several HD-MEAs were used in parallel to speed up experiments. The obtained results are shown in Figure 3C. All current stimulation events delivering charges of up to 3 pC were successful.

As in the case of voltage stimulation, an overlay of the spatial distribution of spontaneous-activity EAP signals and the current stimulation-induced EAP was used to confirm the identity and successful stimulation of the targeted neurons. Spontaneous and stimulated EAPs match temporally, spatially and amplitude-wise (Figure 3D). Additionally, readout electrodes very close to the stimulation electrode also provided clearly detectable signals. The result in Figure 3D can be compared to Figure 2D for voltage mode, where an amplitude comparison was not possible as a consequence of the large artifact.

To summarize, the most efficient stimulation in voltage mode can be achieved by using monophasic cathodic and biphasic anodic-cathodic waveforms with a duration of 100 μs per phase. In current mode, biphasic and triphasic waveforms show the same efficacy, but the biphasic waveform is shorter than the triphasic waveform, which facilitates EAP readout. Durations of 18–20 μs per phase have proven to be efficient (Supplementary Figure 3). Additionally, we noticed that artifact amplitudes are larger for monophasic voltage stimulation and biphasic current stimulation waveforms. In the case of the commonly used voltage stimulation mode, a biphasic anodic-cathodic waveform should be used, as it represents a good combination of stimulation efficacy and artifact duration.

### Impedance Measurements to Compare Voltage and Current Stimulation

As mentioned in Section 3.2, both voltage and current stimulation are widely used to stimulate neuron. Voltage stimulation offers the advantage to reliably obviate Faradaic processes by precisely controlling the electrode voltage and keeping it significantly below 0.8–1 V to obviate water electrolysis and cell and electrode damage (Weiland et al., 2002; Wagenaar et al., 2004). However, the injected charge cannot be controlled and is a function of the electrode impedance. On the other hand, the voltage cannot be controlled upon using current stimulation, so that high electrode voltages can occur in case of high impedance, which may entail unwanted electrochemistry, tissue damage, or electrode degradation. Yet, the charge delivered by the electrode (not the charge path in the preparation) can be precisely controlled, and potentially short current stimulation durations entail short artifacts and fast recovery to baseline values.

To better compare efficacies and differences of current- and voltage-controlled stimulation modes in depolarizing neuronal membranes (from –70 to –55 mV) and evoking APs, it is necessary to also consider the delivered charge. To this end, we established a method to determine electrode impedances, so that charge injection of voltage and current pulses could be calculated and compared.

**FIGURE 4:**
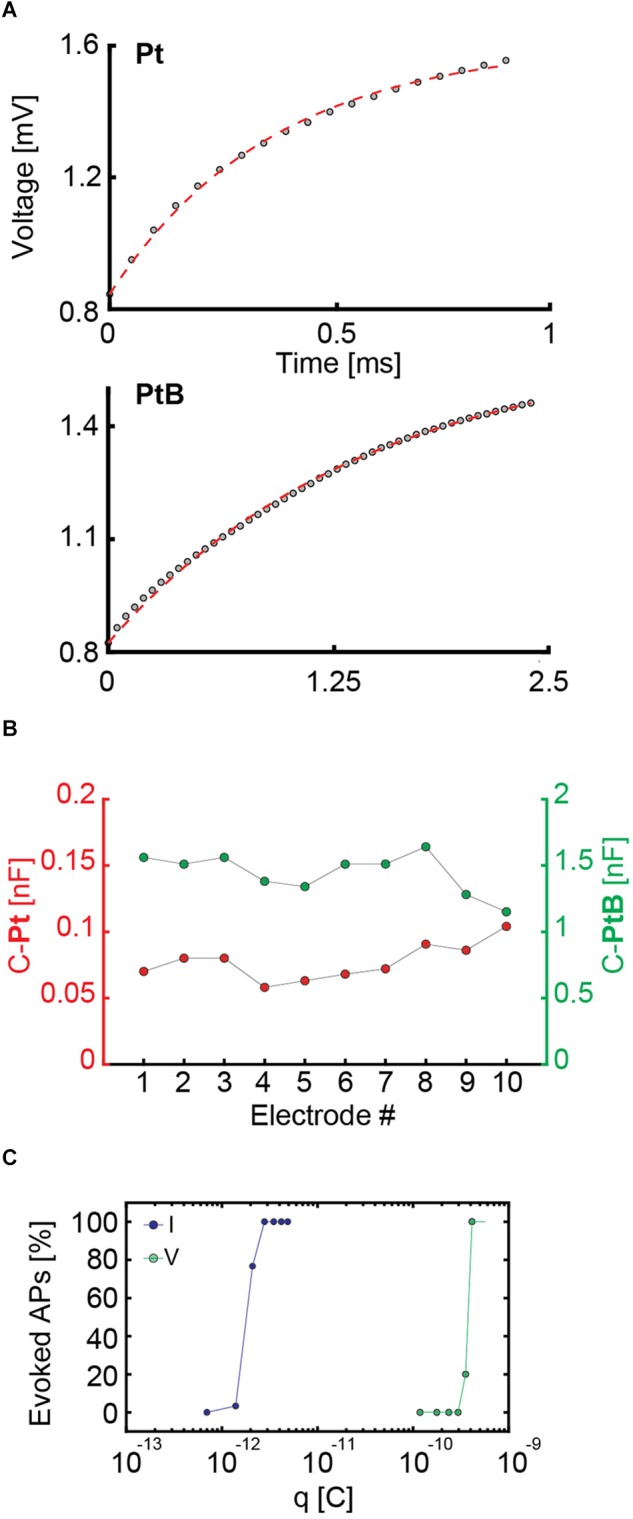
**(A)** Fitting of an electrode model to the experimental data of measured voltage values upon applying a current stimulus to an electrode. For bright Pt (Pt), a current stimulation was performed with a biphasic anodic-cathodic waveform with an amplitude of 140 nA, a duration of 1 ms per phase and a readout amplifier gain of 2. For Pt-black (PtB) the amplitude was 560 nA, the duration 2.5 ms per phase, and the readout amplifier gain was equal to 2. The different waveforms durations and amplitudes are due to the readout channel saturation in case of bright Pt as a consequence of the higher impedance. In both cases, only the first half of the waveform, i.e., the positive part was used for the fits and is displayed. **(B)** Capacitance values of 10 Pt and PtB electrodes are presented. The values were computed as a result of the fitting in panel **A** by setting the capacitance as an unknown value. **(C)** Charges required for efficient voltage and current stimulation of the same neuron. For current stimulation, the waveform had a duration of 20 μs per phase, while the duration for voltage stimulation was 100 μs per phase. The stimulation protocol included 30 repetitions of every stimulation amplitude in a randomized manner. For current stimulation, the charge was computed as *q = I* **×** *t*, i.e., the product of applied current and time. For voltage stimulation, the charge was computed as *q = C* **× Δ***v*, i.e., the product of the computed capacitance and the voltage change upon stimulation.

We applied a biphasic current pulse to an electrode and used a low gain (*G* = 2) for reading out the voltage signals of the same electrode (see Paragraph 2.7). We then fitted the voltage readout from the stimulating electrode with a theoretical electrode model (Supplementary Figure 2 and Figure 4A) by using only the first half of the biphasic anodic-cathodic waveform. The charge transfer resistance, *Rct*, and the double layer capacitance, *Cdl*, were kept as unknown values. After examining (*n* = 10) bright Pt and Pt-black electrodes, we found that the *Cdl* was 0.077 ± 0.0138 nF for bright Pt electrodes and 1.44 ± 0.15 nF for Pt-black electrodes (Figure 4B). The results were obtained with an electrode area of 5 × 9 μm^2^.

To extend these results to a larger number of electrodes, we applied a sine-wave stimulation to 26,400 electrodes and proved that their impedance (in terms of voltage readout) was homogeneous over the array (see histograms in Supplementary Figure 5). This allowed us to use the mean capacitance value of 1.44 nF for Pt-black to compare current and voltage stimulation. Based on the obtained capacitance values, we calculated the charge associated with voltage stimulation and compared it to current stimulation for the same neuron (Figure 4C). We used biphasic waveforms in both modalities, with a duration of 20 μs per phase in current mode and 100 μs per phase in voltage mode. We used a randomized stimulation protocols including 30 repetitions of every stimulation signal amplitude. Using the common capacitor formula C = $\frac{q}{\Delta v}$, we found that, for the very same neuron, the charge delivered through voltage stimulation is two orders of magnitude larger than the one needed to achieve the same results or stimulation efficiency in current stimulation mode (Figure 4C). Our results show that current stimulation, characterized by a constant rate of charge injection, displays higher efficiency in eliciting neuronal responses with respect to voltage stimulation, which is characterized by an exponential decrease in charge injection (see also Supplementary Figure 1). Results of 3 more neurons confirm the same orders of magnitudes and charge differences for current and voltage stimulation (Supplementary Figure 6). The stimulation efficacy in current and voltage mode is also largely depending on the stimulation buffer implementation (Ballini et al., 2014).

### Multi-Electrode Stimulation

An array of densely distributed electrodes enables to apply different stimulation configurations, either by using the standard single-electrode stimulation approach, or by selecting several electrodes at the same time for applying signals or for grounding. Normally, all unused array electrodes are left floating and do not have a defined potential. The use of neighboring electrodes as stimulation and reference or ground electrodes can produce a locally larger electric field strength, which, in turn, could lead to lower voltages required for stimulation in voltage mode. Moreover, it is possible to stimulate with opposite-sign waveforms on adjacent electrodes to reduce and limit stimulation artifacts.

**FIGURE 5:**
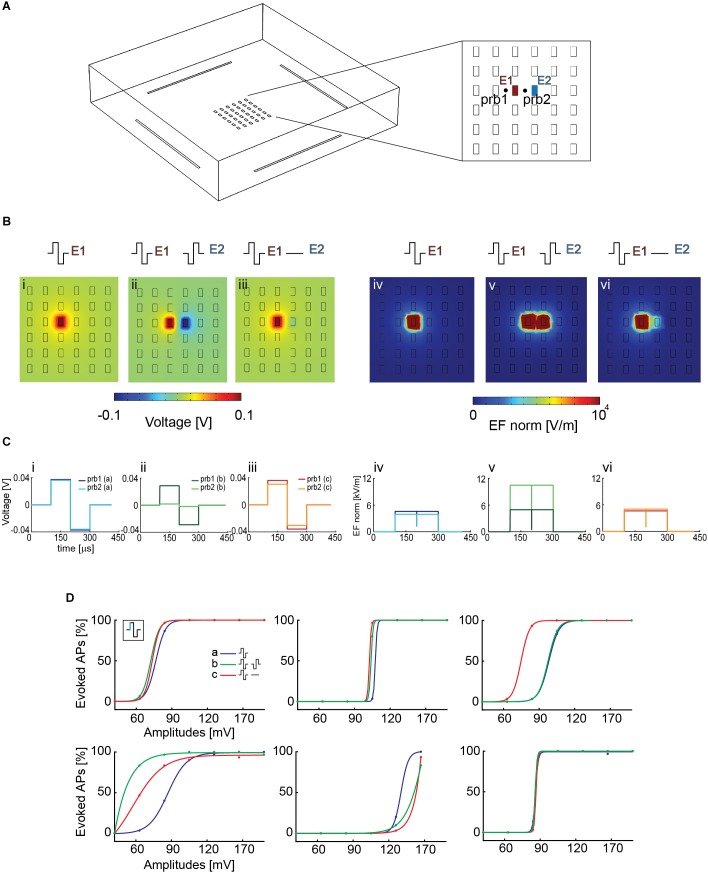
**(A)** Simplified HD-MEA geometry for COMSOL simulations: 36 planar electrodes at a pitch of 17.5 μm and featuring an area of 5 × 9 μm^2^; 4 reference electrodes were placed at the borders of the array. E1 and E2 denote the electrodes that were used for the simulations in panel **B**. prb1 and prb2 denote two probe locations, at the right and at the left side of electrode E1, that were used to compute the voltage and the electric-field values for the three configurations in panel **B**. The two probe locations were chosen so as to compare the effect of using a second array electrode. **(B)** The simulation was performed in voltage mode, and an amplitude of ±100 mV was used. The voltage and electric field distributions after stimulating with one electrode against a global reference electrode in solution are represented in panels (i) and (iv), those for a stimulation with two neighbored electrodes with synchronized waveforms of opposite signs are represented in panels (ii) and (v), and those for applying a biphasic waveform to one electrode, while the neighboring electrode was grounded are represented in panels (iii) and (vi). The electric field (EF norm) was calculated as $\sqrt{E_{\text{x}}^{2}+E_{\text{y}}^{2}+E_{\text{z}}^{2}}$. **(C)** Voltage (i, ii, iii) and electric-field (iv, v, vi) distributions at the probe locations in the respective configurations (i–vi) of panel **B**. The voltage drop, which is responsible for the AP initiation by electrical stimulation, is comparable in all the cases, except for the two-electrode stimulation (ii), where it is somewhat decreased. The electric field is stronger in the case of the two-electrode stimulation (v). **(D)** Stimulation with a biphasic voltage waveform of six different neurons using the three configurations simulated in COMSOL in panel **B**. There were differences in the voltage required to evoke activity in the different neurons (range between 60 and 120 mV), however, there were no major differences for using the three stimulation scenarios explained and displayed in panel **B**.

Using COMSOL Multiphysics, we simulated the voltage and electric field distribution on the array for different candidate electrode configurations to see if an increase in electric field strength could increase neuron stimulability and decrease the artifact lateral extension. Three configurations were selected (Figure 5A,B): (i, iv) stimulation with a biphasic voltage waveform (±100 mV) through one electrode against a global reference electrode in solution; (ii, v) stimulation with two neighboring electrodes, using biphasic voltage waveforms (±100 mV) with opposite signs; (iii, vi) stimulation with a biphasic waveform (±100 mV) applied to one electrode, while the neighboring electrode was grounded. All other array electrodes were left floating.

To assess if one of the two chosen configurations with local ground or opposite-sign stimulation signal could improve stimulation efficiency by entailing higher local voltage drops or electric-field strengths, we simulated two probe locations at the right and left side of electrode E1 (Figure 5A), at a height of 1 μm above the electrode plane. For configuration (i), one stimulation electrode against a global reference electrode, the voltage drop left and right of the electrode was identical, because the global reference electrode is far away and positioned outside the electrode array. In configuration (iii), the grounded neighboring electrode slightly modified the voltage drop at the right probe location. In configuration (ii), however, the application of a signal to the neighboring second electrode induced a voltage drop decrease at the location of the right probe, as a result of the applied opposite-sign voltages (Figure 5C). However, the electric field between the two electrodes was increased (v) (Figure 5C). Nevertheless, looking at the voltage distribution around the stimulation electrodes suggests comparable results for all configurations, with even a possible decrease in stimulation efficacy for axons that would run through the center region between the two electrodes with opposite-sign waveforms.

To verify the simulation, we then tested the efficacy of these three configurations in evoking APs in neuronal cultures on the HD-MEA. We stimulated six different neurons, after having determined the most reliable electrodes in evoking APs at the respective AISs. First, a randomized voltage stimulation protocol was applied to one electrode, using the global reference electrode at the periphery of the array. A biphasic anodic-cathodic voltage stimulation waveform, evidenced to be efficient (Section 3.2), was used with a phase duration of 100 μs and an amplitude range between 40 and 160 mV. We then repeated the same stimulation protocol by using a grounded reference electrode close to the stimulation electrode to increase the electric field. Finally, we used two electrodes delivering opposite waveforms for stimulation, which further increased the local electric field and, additionally, reduced the lateral extension of the artifact (Supplementary Figure 7). In all the modalities, also the reference electrodes at the sides of the array were left connected. We found differences in the voltage required to evoke activity in the different neurons (range between 60 and 120 mV, Figure 5D), however, we did not find major differences for using the three stimulation scenarios explained, simulated and displayed in Figure 5B,C.

This experimental result is in line with the simulations in Figure 5C, which shows comparable extracellular voltage levels for all three configurations. Consequently successful stimulation and APs initiation is not much influenced by applying the three different configurations.

### Stimulability Across Cell Development Increases in Early Stages of Neuronal Growth and Development

**FIGURE 6:**
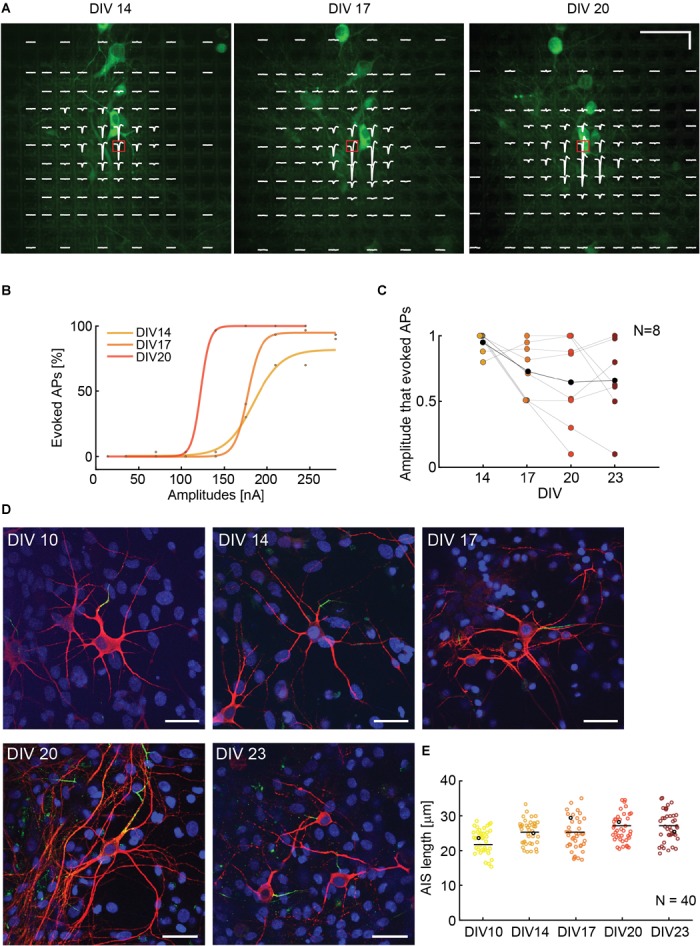
**(A)** Superimposed fluorescence image and electrical footprint of the same neuron at DIVs 14, 17, 20. The stimulation electrodes used for the analysis are indicated with a red box. The stimulation electrode changed position with the neuron movements during the experiment. The signals recorded on the different electrodes are displayed in white (electrical footprint). Horizontal scale bar: 50 μm, vertical scale bar: 100 μV. **(B)** Current stimulation activation curves of the neuron in panel **A** over time (DIVs). The stimulation amplitudes necessary to evoke APs decreased between DIV 14 and 20. **(C)** Stimulability experiments for *N* = 8 neurons. For every neuron, the stimulation amplitudes were normalized to the highest amplitude that was necessary to evoke APs 90% of times over 30 repetitions during the DIVs 14–23. The stimulability varied from neuron to neuron. We observed a trend of decreased stimulation amplitudes between DIV 14 and 20 that then stabilization between DIV 20 and 23. The average relative stimulation current amplitude is represented with black dots. **(D)** Sample microscopy stainings showing fixed neurons at DIV 10, 14, 17, 20, 23. The nuclei are represented in blue, the neurons in red and the AISs in green. The 5 neurons are represented as black dots in panel **E**. Scale bar 35 μm. **(E)** AIS lengths and length distribution over time: DIVs 10, 14, 17, 20, 23. The average length tends to slightly increase over time *in vitro* (*N* = 40).

To assess if the ability to stimulate neurons is correlated to cell culturing time and AIS growth, we observed and stimulated single neurons during different DIVs. The experimental time points were 14, 17, 20, and 23 DIV. We used NeuroFluor NeuO to do live-staining of neurons (Figure 6A). To ensure staining effectiveness, we repeated the staining before every experiment. Isolated cells were identified and three to six stimulation electrodes were used for stimulation. We chose the most efficient stimulation electrode, which was the electrode with the highest extracellular voltage readout (AIS), to execute the stimulation protocol. We used a randomized current stimulation protocol to avoid neuronal adaptation (Grubb and Burrone, 2011). The use of current stimulation was motivated by the reduced artifact (Paragraph 3.1) and the more reliable AP readout (Paragraph 3.2). A biphasic anodic-cathodic waveform was used with a duration of 20 μs per phase. We observed that, over 10 DIVs, the neurons moved by a maximum of two electrode distances, which is equal to 35 μm. In case that the neurons moved by one electrode distance or more, the stimulation electrode was also changed. If the neurons did not move, the most efficient stimulation electrode remained the same (Figure 6A). The movement was evaluated by using the electrical “image” and simultaneous upright confocal imaging. We monitored the soma position with respect to the stimulation electrode position during the experiment days. The results show that the stimulation amplitudes, required to evoke APs, decreased during the first half of the experiment (from DIV 14 to DIV 20), and stabilized around DIV 20 and 23 (Figure 6B,C). An increase in stimulation amplitudes was mostly due to neuron movement and change of the relative position with respect to the stimulation electrode as evident, e.g., in Supplementary Figure 8 for day 20. We also recorded and compared the EAPs of spontaneous neuronal activity over the different DIVs so as to ensure the identity of the respective neurons. To verify if the increase in stimulability was correlated with an AIS growth, we stained neurons at DIV 10, 14, 17, 20, and 23 and we computed the length of *N* = 40 AIS. We found that there is the tendency of an AIS-average-length increase with increasing DIVs (Figure 6D).

## Discussion

In this study we showed that it is possible to selectively and reliably stimulate individual neurons by applying current and voltage pulses through the 26,400 electrodes of an HD-MEA. We tested different stimulation waveforms, durations and amplitudes in voltage and current mode. We demonstrated that we were able to stimulate individual neurons by combining high-density recording of single-neuron action potentials with immunostaining and confocal microscopy. Previous studies (Wagenaar et al., 2004) reported that, in voltage mode, the biphasic anodic-cathodic waveform was most efficient for selective stimulation, followed by the cathodic-anodic one. We found that the most efficient stimulation waveform is the monophasic cathodic waveform, followed by the biphasic anodic-cathodic, by comparing the V_pp_ for the same pulse width (Figure 2). It is, however, important to note that we used significantly lower stimulation voltages (80 mV_pp_), as we could target the most sensitive region of a neuron, the AIS, for stimulation. Moreover, we used much smaller electrodes with respect to the 30 μm diameter electrodes in the study by Wagenaar et al. (2004). In current mode, we confirmed that the triphasic waveform is an efficient stimulation signal that produces a comparably smaller artifacts than the biphasic one (Grosberg et al., 2017). In comparison to previous findings, we were able to improve selectivity due to the high electrode density and the inherent possibility of directed targeting of neurons and their AISs (Radivojevic et al., 2016). We, hence, could stimulate with comparably low charges of a few picoCoulombs (Figure 3). Moreover, the small electrodes provided sufficient stimulation charge density also for small applied voltages.

In comparing current and voltage stimulation parameters for our HD-MEA, we found that a waveform duration of 100 μs per phase was required to efficiently evoke EAPs in voltage mode. In current mode, instead, a duration of only 20 μs per phase was required, which entailed a shorter artifact duration. However, it needs to be mentioned that the possible shorter signal duration in current mode was also a consequence of the settling time of our stimulation buffers. According to our measurements, current stimulation is the preferable stimulation modality, which is in line with reports in literature (Grosberg et al., 2017; Fan et al., 2019). By using current stimulation it was possible to use already the next neighboring electrodes, at 17.5 μm pitch from the stimulation electrode, for EAP recording (Figure 1, 2). Owing to the high spatial resolution and dense electrode packing of the array, it was possible to read out electrical activity at the cell soma while stimulating the AIS of the very same cell, so that it was possible to accurately estimate stimulation success and efficiency.

Based on our experiments, we estimated current stimulation to be more efficient than voltage stimulation in evoking APs using our HD-MEA. To further test this assumption, we determined the electrode impedances by measuring and modeling the voltage readout upon applying current stimulation (Franks et al., 2005). The fit of the measurement data to the model returned capacitance values of ∼1.4 nF for Pt-black and ∼0.07 nF for bright Pt electrodes. We used these capacitance values to compare voltage and current activation curves, and found that the charge needed to evoke APs in current mode is, indeed, by two orders of magnitude lower than in voltage mode (Figure 4). The stimulation efficacy in current and voltage mode is also depending on the stimulation buffer implementation (Ballini et al., 2014).

We then compared different electrode configurations, which provided increased electric field strengths, as compared to using a single electrode against a global reference electrode in solution. As shown in Figure 5D, the stimulation efficacy, however, was found to be in the same range for different neurons.

Capitalizing on the short artifact duration upon using current mode, we finally studied neuron stimulability during cell development and growth. Our hypothesis was that the AIS development over time would increase neuron stimulability. We combined live stainings and electrical recordings/stimulation to follow neuronal development over several days. The most challenging procedure during the experiments was to identify more or less isolated neurons on the array and in the culture and to then track them over several days during the experiments. Several neurons had to be discarded as a consequence of cell death during the experiments or because we could not track them over the experiment time. Nevertheless, we observed a decrease in stimulation amplitudes to evoke APs, which was correlated to AIS growth in length (Figure 6).

In summary, this work presents a comprehensive study on electrical stimulation with microelectrodes of HD-MEAs and shows ways to realize single-neuron stimulation. Selecting optimal stimulation parameters could prove to be powerful for other *in vitro* applications, such as the control of neural-network bursting through electrical stimulation (Wagenaar et al., 2005), or for *ex vivo* stimulations, for example, in retinal preparations or brain slices. We think that *in vivo* stimulation methods (epiretinal implants) could also benefit from findings of this paper in order to implement targeted stimulation of individual neurons. The delivered charges to depolarize neuronal membranes amounted to 0.02 pC/μm^2^ with our HD-MEA, while, for example, retinal implants currently work with 3.5 pC/μm^2^ (Ahuja et al., 2013). A small size of electrodes and their dense packing may prove beneficial to stimulate neurons and could improve stimulation accuracy of prosthetic implants while enabling lower power consumption.

## Data Availability

The datasets generated for this study are available on request to the corresponding author.

## Author Contributions

SR, MF, JM, VV, UF, and AH performed experimental design. SR and CM performed experiments and data analysis. MF, JM, and VV provided technical support. SR and AH wrote the manuscript. MF, CM, VV, JM, and UF reviewed the manuscript. AH supervised the project.

## Conflict of Interest Statement

MF, JM, and UF are co-founders of MaxWell Biosystems AG, which commercializes HD-MEA technology. The remaining authors declare that the research was conducted in the absence of any commercial or financial relationships that could be construed as a potential conflict of interest.
