# Supplementary material for: Single-Cell Electrical Stimulation Using CMOS-Based High-Density Microelectrode Arrays
Source: Front Neurosci. 2019 Mar 13;13:208. doi: 10.3389/fnins.2019.00208 (PMC6424875; doi:10.3389/fnins.2019.00208)
Supplement: Supplementary file 1 [file Data_Sheet_1.docx]

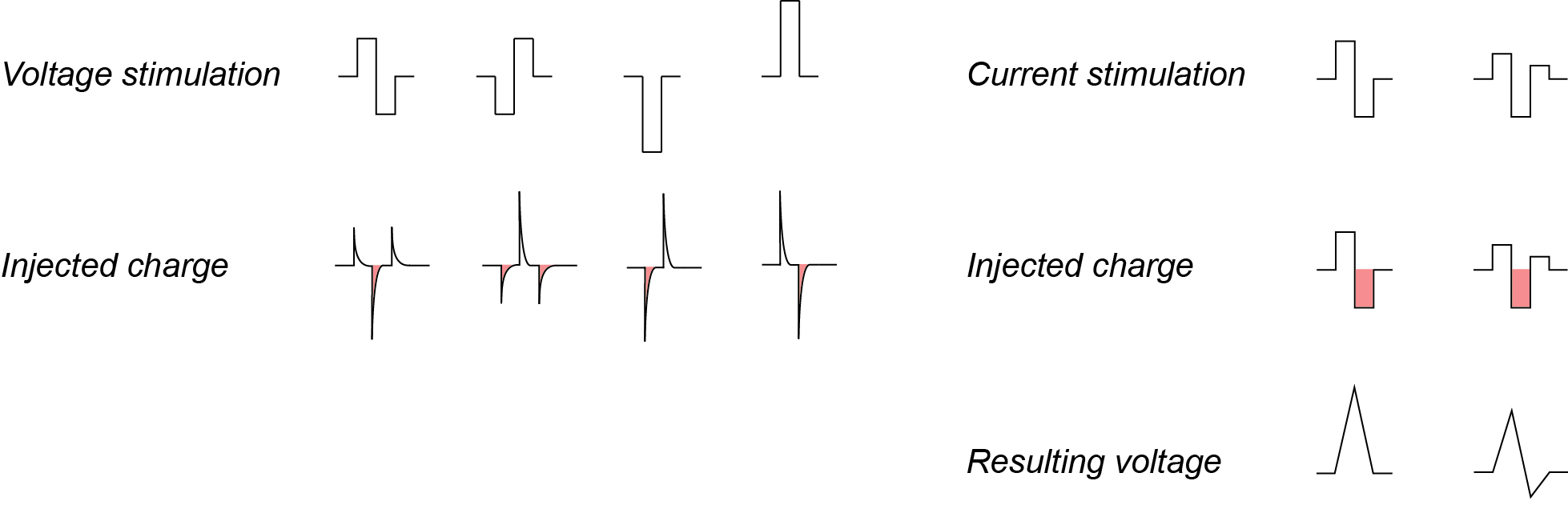


Supplementary Figure 1

Stimulation pulses used for voltage (left) and current (right) mode are shown in the top row. Below a representation of the injected charges in voltage (left) and current (right) mode. The negative charge that evokes APs is displayed in red. In voltage mode, the charge features exponential decays and is a function of the electrode impedance, while it stays constant in current mode.


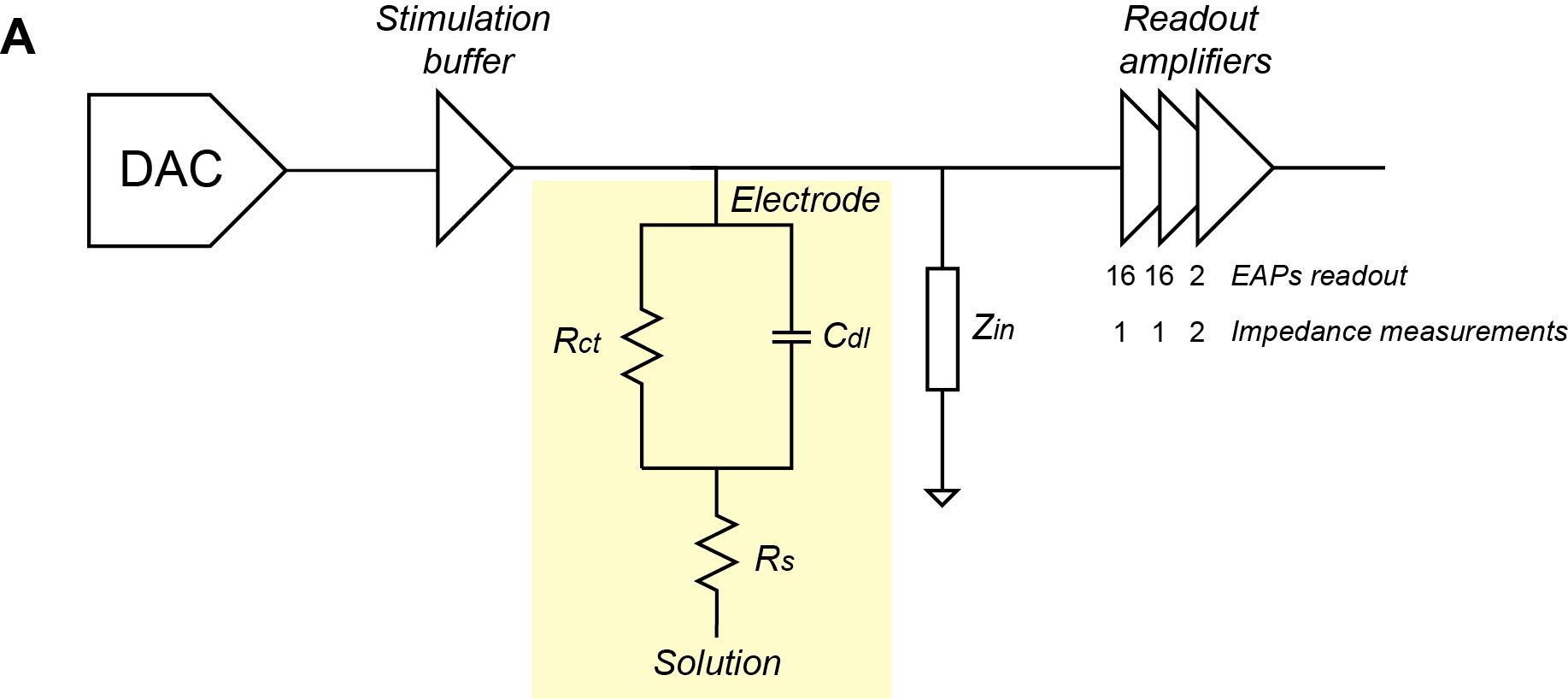


Supplementary Figure 2

Equivalent circuit model of the stimulation and readout channel. In yellow, the electrode equivalent circuit including the double-layer capacitance $C_{dl}$ and the charge transfer resistance $R_{ct}$ is shown along with the solution resistance $R_{s}$. $Z_{in}$ represents the input resistance to the readout amplifiers. To read out extracellular APs, a gain of 512 was used (3 gain stages: 16×16×2). For impedance measurements, instead, the first two amplifier stages were bypassed, while the third one was set to a gain of 2. The low gain enabled a voltage readout on the stimulation electrode.


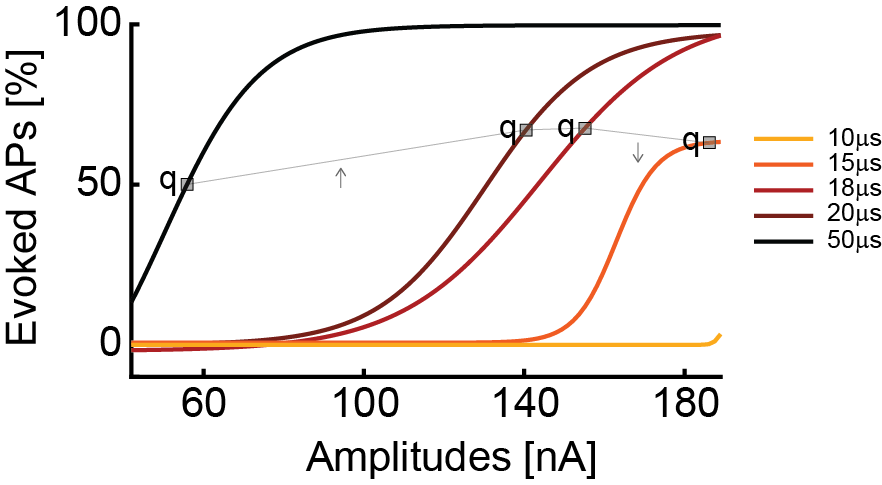


Supplementary Figure 3

Activation curves as a result of current stimulation with a biphasic waveform. The label *q* indicates the points, where the delivered overall charge is the same. While the efficacy is almost the same for 20 and 18 µs phase duration, for 15 µs it starts to decrease. This behavior can be explained with the fact that the stimulation buffers cannot efficiently deliver signals of very short duration.


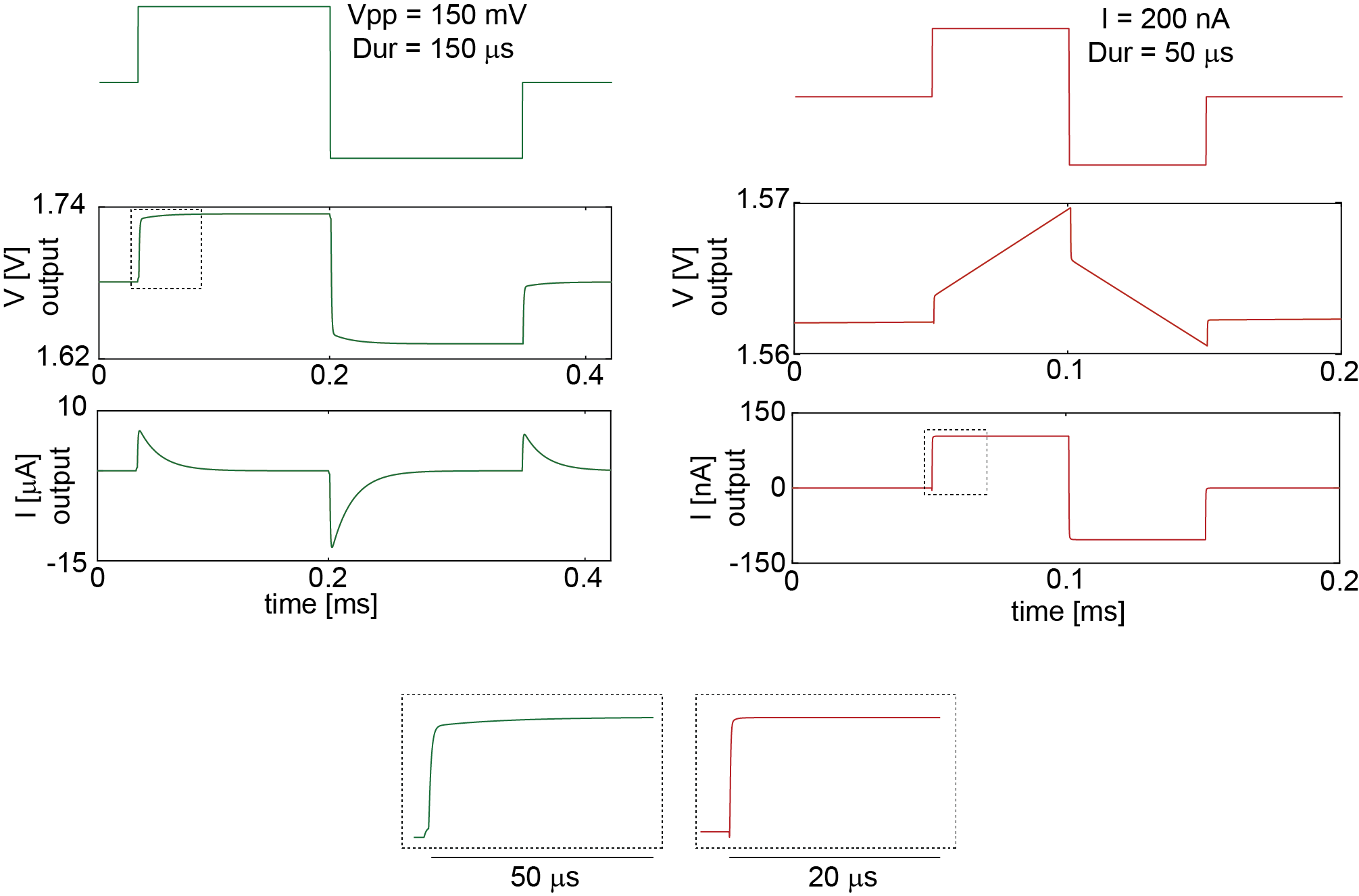


Supplementary Figure 4

Simulated voltage (green) and current (red) output signals of the stimulation buffers. The settling time of the stimulation buffers in voltage mode is ~50 µs to reach the applied voltage value, while in current mode, the applied value is reached almost instantaneously. The difference is due to the different stimulation buffers’ design. The simulations were performed considering an equivalent electrode model with $C_{dl}$ = 1.5 nF, $R_{ct}$ = 100 MΩ and $R_{s}$ = 10 kΩ.


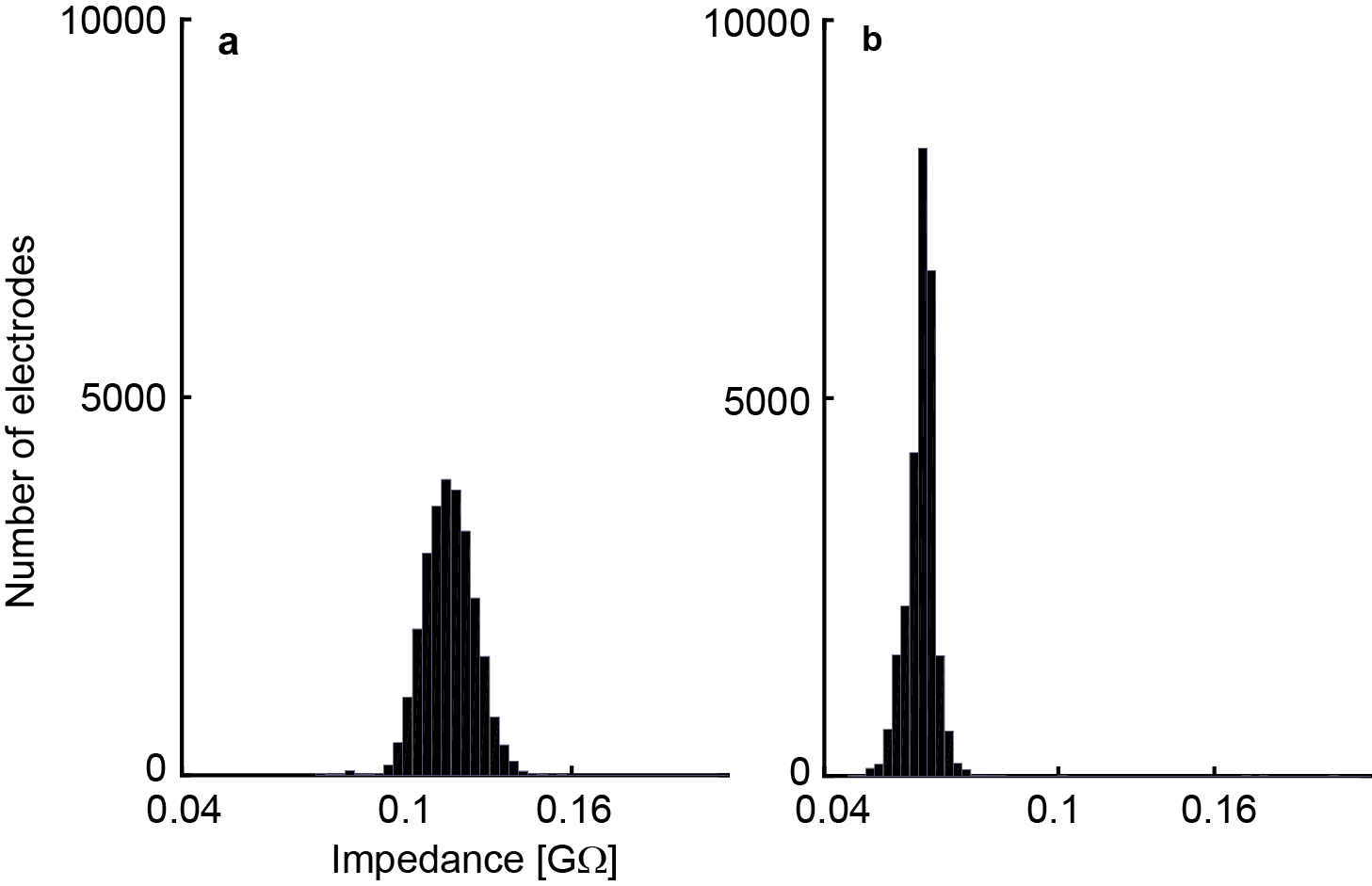


Supplementary Figure 5

Histograms of the impedance distribution of all 26’400 electrodes to demonstrate the impedance homogeneity (a bright Pt, b Pt-black). A sinusoidal waveform was used for stimulation.


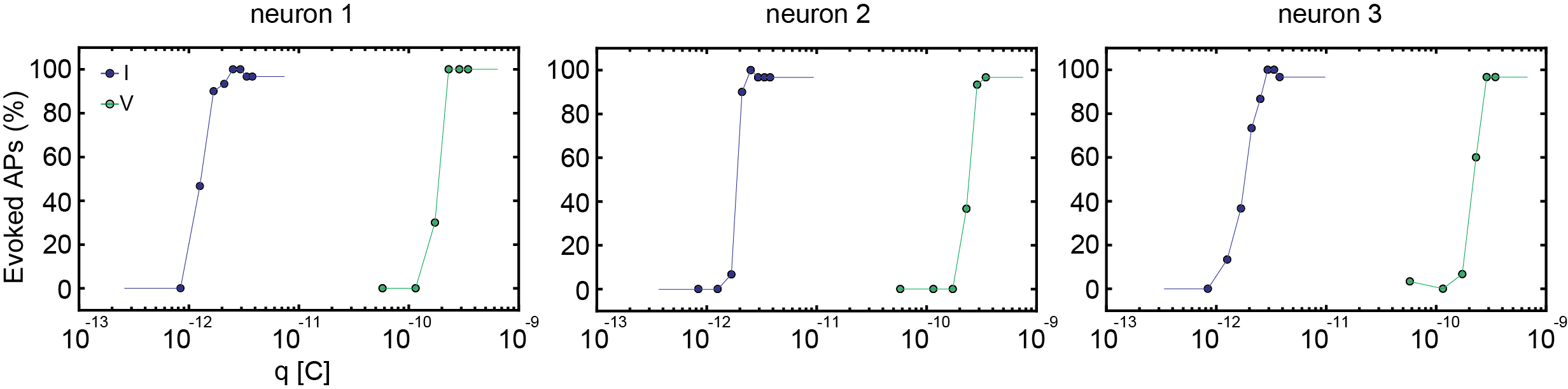


**Supplementary Figure 6**

Charges required for efficient voltage and current stimulation of three different neurons. For current stimulation, the waveform had a duration of 20 µs per phase, while it was 100 µs per phase for voltage stimulation. The stimulation protocol included 30 repetitions of every stimulation-signal amplitude in a randomized manner.

**
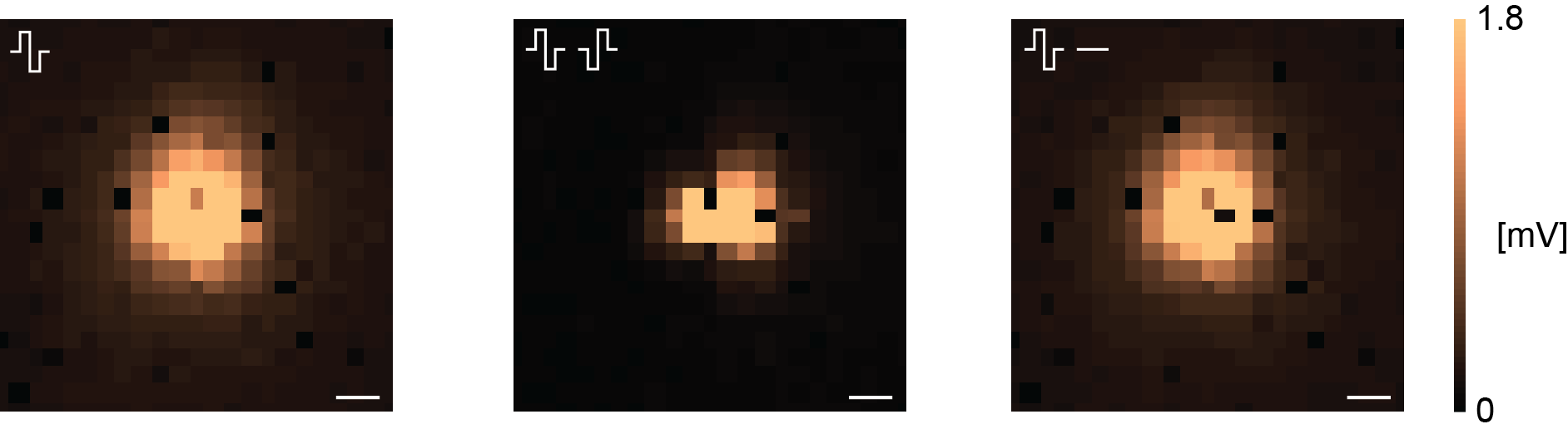
**

**Supplementary Figure 7**

Recordings from high-density blocks of electrodes to compare the stimulation artifact spread in cell-culture medium. Three configurations were used: one stimulation electrode against a global reference electrode in solution (left), two neighboring electrodes with opposite-sign waveforms (center), and one stimulation electrode, while the neighboring electrode was grounded (right). The configuration with two electrodes delivering waveforms of opposite signs seemingly produced the smallest artifact on the array. The artifact was computed as the peak-to-peak voltage readout of every electrode. The measurements were done in growth medium. The voltage stimulation amplitude was 140 mV, and the duration 100 µs per phase. N=1. Scale bar 50 µm.


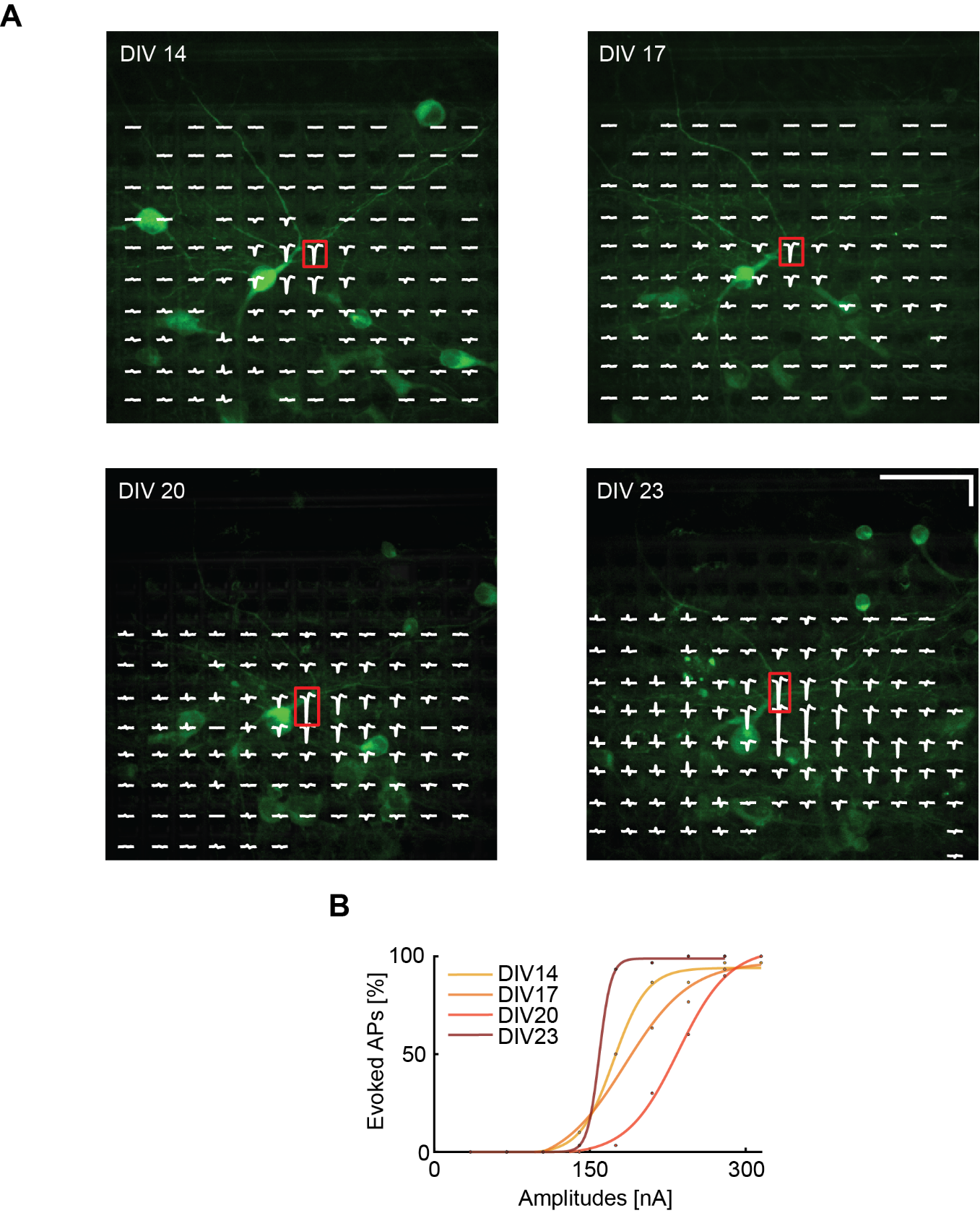


**Supplementary Figure 8**

**(A)** Superimposed fluorescence image and electrical footprint of another neuron at DIVs 14, 17, 20, 23. This neuron significantly moved during the initial measurements between DIVs 14 and 20 so that the stimulation threshold increased. The stimulation electrodes used for the analysis are indicated with a red box. The stimulation electrode remained the same during the whole experiment. The signals recorded on the different electrodes are displayed in white (electrical footprint). Horizontal scale bar: 50 µm, vertical scale bar: 250 µV. **(B)** Current stimulation activation curves of the neuron in A over time (DIVs). The stimulation efficacy varied and increased between DIV 20 and 23. The initial decrease is due to neuron movement.
